# Investigation of SERS and Electron Transport Properties of Oligomer Phenylacetyne-3 Trapped in Gold Junctions

**DOI:** 10.3390/nano12030571

**Published:** 2022-02-07

**Authors:** Ziyu Liu, Tingting Hu, Muwafag Osman Adam Balila, Jihui Zhang, Yujin Zhang, Wei Hu

**Affiliations:** 1 School of Chemistry and Chemical Engineering, Qilu University of Technology (Shandong Academy of Sciences), Jinan 250353, China; liuzy0625@163.com (Z.L.); hutingting_1981@163.com (T.H.); moufagbalila533@gmail.com (M.O.A.B.); 2 Technology College of Chemical Engineering, Qingdao University of Science, Qingdao 266061, China; 3 School of Materials Science and Engineering, Qilu University of Technology (Shandong Academy of Sciences), Jinan 250353, China; beckhamzjh@163.com; 4 School of Electronic and Information Engineering, Qilu University of Technology (Shandong Academy of Sciences), Jinan 250353, China

**Keywords:** molecular junction, SERS, electron transport

## Abstract

Molecular junctions hold great potential for future microelectronics and attract people’s attention. Here, we used density functional theory calculations (DFT) to investigate the surface-enhanced Raman spectroscopy (SERS) and electron transport properties of fully π-conjugated oligomers (phenylacetylene)-3 (OPE-3) trapped in gold junctions. The effects of charge injection, an applied electric field, and molecular deformation are considered. We found that a new Raman peak located at around 1400 cm^−1^ appears after the injection of a charge, which agrees well with the experiment. The external electric field and configurational deformation hardly affect the Raman spectra, indicating that the electronic rather than the geometrical structure determines the Raman response. Nonequilibrium Green’s function (NEGF) calculations show that both the rotation of the benzene groups and an increased electrode distance largely reduced the conductivity of the studied molecular junctions. The present investigations provide valuable information on the effect of charging, electric field, and deformation on the SERS and conductivity of molecular junctions, helping the development of molecular devices.

## 1. Introduction

The last 30 years have witnessed the rapid development of molecular junctions and a wide variety of functional molecule devices (diodes, resistors, switches, sensors, LEDs, etc.) have been proposed [1,2,3,4,5,6]. Single molecular junctions can be identified as a dual interface material because of the metal–molecule–metal structure [7,8,9]. The complicated contact introduces the hybrids of the molecular orbitals with the metal [10,11,12,13,14]. Studying the unique electronic characteristics of a single-molecule electronic device can largely help us to understand its rectification, switching, and magnification functions [15,16,17,18,19,20].

Surface-enhanced Raman scattering (SERS) is a suitable method for characterizing the vibrational spectra of molecular junctions [21,22,23,24,25,26,27]. In the previous work, we studied the SERS characterization of p-terphenyl-4,4″-dithiol and its 2,2′,5′,2″-tetramethylated analogue in gold junctions to investigate the molecular deformation mechanism [28]. We found that both injecting charges and applying electric fields can introduce the deformation of trapped molecules. However, only the SERS response with an applied electric field can reproduce the experiment.

Recently, Bi et al., constructed gold junctions using a π-conjugated oligomer phenylene acetylene-3 (OPE-3) molecule [29]. They investigated the surface-enhanced Raman spectroscopy (SERS) and charge transport properties. They found that applying a voltage bias of 0.5 V introduced new Raman peaks located at around 1400 cm^−1^. Even though they had proven that charging is the underlying mechanism of the changes in the Raman spectra, a systematical theoretical simulation is still highly desired to investigate the effects of charging, electric fields, and molecular deformation on the Raman response and charge transport properties.

## 2. Materials and Methods

Using the hybrid B3LYP functional and def2tzvp basis sets incorporated in the Gaussian 09 software package [30,31], we examined the effects of charge injections and external electric fields on the conformations, electronic structures, and SERS responses [32,33].

**Figure 1:**
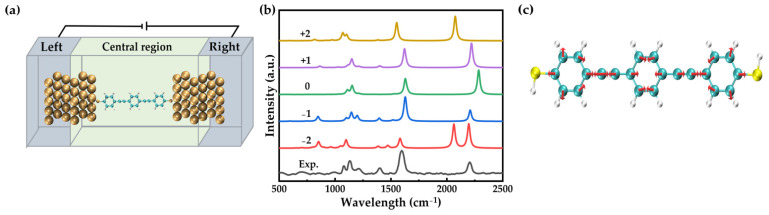
(**a**) Schematic diagram of oligomer phenylene acetylene-3 (OPE-3) trapped in gold junctions. (**b**) Simulated Raman spectra of the OPE-3 molecule with five different injected charges (−2, −1, 0, +1, +2). (**c**) Raman active vibrational modes at 1400 cm^−1^.

The uniform electric field is applied using the keyword “Field” implemented in the Gaussian package. The Atomistix ToolKit (ATK) package [34] was applied to study the charge transport properties using the nonequilibrium Green’s function (NEGF) method in combination with density functional theory (DFT). As shown in Figure 1a, a unit cell (3 × 3) of Au (111) was selected to model the electrodes [25,35]. The molecular devices are divided into three parts: the extended molecule (containing six Au layers and molecules) and two semi-infinite gold electrodes. The Perdew–Burke–Ernzerhof (PBE) formulation of the generalized gradient approximation (GGA) was used as the exchange correlation function until the forces on each atom became less than 0.05 eV/Å. The mesh cutoff energy of 300 Ry was selected to achieve a balance between the calculation efficiency and the accuracy. The Monkhorst Pack k-point sampling of the Brillouin zone measured 1 × 1 × 1.

## 3. Results

We first studied the influence of charging on the Raman spectra. It is well known that gold electrodes exhibit good conductance and trapped molecules, poor conductance. As a result, most of the voltage was applied on the trapped molecule (OPE-3) and the OPE-3 molecule, rather than the gold electrodes, can be easily charged. The geometries of the OPE-3 molecule were optimized after the five charges of −2, −1, 0, +1, and +2 were injected. Good planar conjugation was found, regardless of the number of injecting charges. The average dihedral between the three benzene groups was found to be 0.1° for the OPE-3 molecule with a charge of +2, +1, 0, −1, and −2, respectively. On the other hand, charging largely affected the frontier orbitals of the OPE-3 molecule. As shown in Figure S1 in Supplementary Materials, the HOMO-LUMO gap for positive and negative OPE-3 molecules decreased compared to the neutral form. The change in the HOMO-LUMO gap, as well as the polarizability, further introduced both absolute and relative changes in the Raman intensities.

As shown in Figure 1b, the Raman spectra of −1 charged molecules was consistent with the experimental spectra. This conclusion was obtained from the following three aspects: (1) The Raman spectrum of negatively charged molecules has a new peak around 1400 cm^−1^ (the coupling mode of the acetenyl-benzene stretching and C-H wagging). (2) The three peaks around 1152 cm^−1^ (the coupling mode of benzene in-plane deformation and C-H wagging) are well reproduced. (3) The relative intensities of the three peaks located at around 1152, 1594 (C-C stretching in the benzene groups), and 2187 cm^−1^ (C-C stretching in the acetenyl groups) can be reproduced adequately. Although the peak location is different from the experiment, such as the peaks at 1152 and 1594 cm^−1^ in the experiment are blue shifted in theory, but it is trivial.

Except for the −1 charged form, the others cannot reproduce the experimental spectrum. For instance, no peak appeared at around 1400 cm^−1^ for the molecules charged at 0 and +2. For the −2 charged form, two peaks located at 2146 and 2284 cm^−1^ appeared, which is poorly consistent with the experimental spectrum. For the +1 charged molecule, only one strong peak appeared near 1152 cm^−1^, which does not agree with the three comparable peaks in the experimental spectrum. Furthermore, the relative intensity of the two peaks located at 1594 and 2187 cm^−1^ is different from the experiment. In our calculations, the vibrational modes are around 1400 cm^−1^, which corresponds to the vibration of single bonds, phenyl rings, and triple bonds, respectively, as shown separately in Figure 1c.

We then studied the effect of the electric field on the Raman spectra. We first considered the effect of the direction of the electric fields on the Raman spectra. As shown in Figure S2 in the Supplementary Materials, we can see that the applied electric field from the direction of X, Y, and Z varies. However, considering the OPE-3 molecule connected with an Au electrode via Au–S bond, here, we focus only on the electric field along the junction.

**Figure 2:**
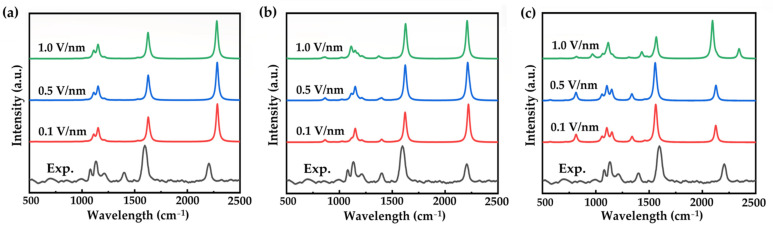
Simulated Raman spectra of a neutral (**a**), positive (**b**), and negative (**c**) OPE-3 molecule with different electric fields.

Here, we applied a uniform electric field of 0.1, 0.5, and 1.0 V/nm on the 0, +1, and −1 charged forms. It is noted that the effect of the electric field on the geometrical structure can be ignored as the OPE-3 molecule maintains its plane structure and well conjugation. As shown in Figure 2, the Raman spectrum of neutral molecules does not change much with varying the electric field and no new peak appears at around 1400 cm^−1^ (as shown in Figure 2a). For the positively charged form (+1), the relative intensities of the peaks located around 1200 cm^−1^ see complicated changes through varying the electric field (as shown in Figure 2b). However, we can see from Figure 2a,b that varying the electric fields hardly changes the whole Raman spectra of the OPE-3 molecule with 0 and +1 charges.

On the other hand, the electric field largely changes the Raman spectra of the negative OPE-3 molecule. As shown in Figure 2c, the peak located at 1400 cm^−1^ shows a blue shift as the electric field increases. Moreover, at 2200 cm^−1^, there is splitting with an increasing electric field. To be specific, peaks at 2184 and 2207 cm^−1^ appear when applying an electric field of 0.5 V/nm. These values are 2095 and 2343 cm^−1^ with an electric field of 1.0 V/nm.

**Figure 3:**
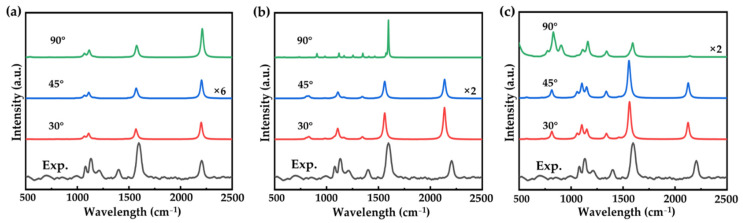
(**a**) Raman spectra of neutral (**a**), positive (**b**), and negative (**c**) OPE-3 molecules with a rotated dihedral of 30, 45, and 90°. “×6” and “×2” indicate the magnification factors of specific Raman spectra compared to that of the configuration with a 0° dihedral (Figure 1b).

It is well known that molecular junctions always show fragility and geometrical variation caused by external solutions and temperatures. To objectively evaluate the potential of OPE-3 molecular junctions as future semiconductors, we studied the dependence of the electric transport properties and SERS spectra on geometrical fluctuations. We first studied how the Raman spectra change with changes to electrode distance. As shown in Figure S3 in the Supplementary Materials, we found that the SERS signals varied little by decreasing or increasing the electrode distance (see Figure S3 in Supplementary Materials for detail). Then, we studied the effect of deformations of the OPE-3 molecule on the SERS spectra. We kept the left and right benzene rings of the OPE-3 molecule in a plane and rotated the middle benzene by 30, 45, and 90°. The calculated Raman spectra are shown in Figure 3a–c. The larger HOMO-LUMO gap in tilting configurations (shown in Figure S4 in Supplementary Materials) make the Raman intensities lower than that of the configuration with a 0° dihedral (Figure 1b). As shown in Figure 3a, no peak appears at around 1400 cm^−1^. Although a weak peak appears at around 1400 cm^−1^ for the positive form with a rotation dihedral of 30°, the relative intensity cannot match the experimental one. The negative form shows a very interesting phenomenon when rotating the middle benzene. As shown in Figure 3c, rotation dihedrals of 30° and 45° bring little change to the Raman spectra. However, the Raman spectra present huge changes with a rotation dihedral of 90°. It is well known that both the electronic and geometrical structure can determine the molecular polarizability, as well as the Raman activities. However, for the well-conjugated OPE-3 molecule, the addition or release of electrons brings a larger change in the polarizability compared to geometrical torsion. From this point of view, we can conclude that the electronic rather than the geometrical structure determines the Raman spectra.

**Figure 4:**
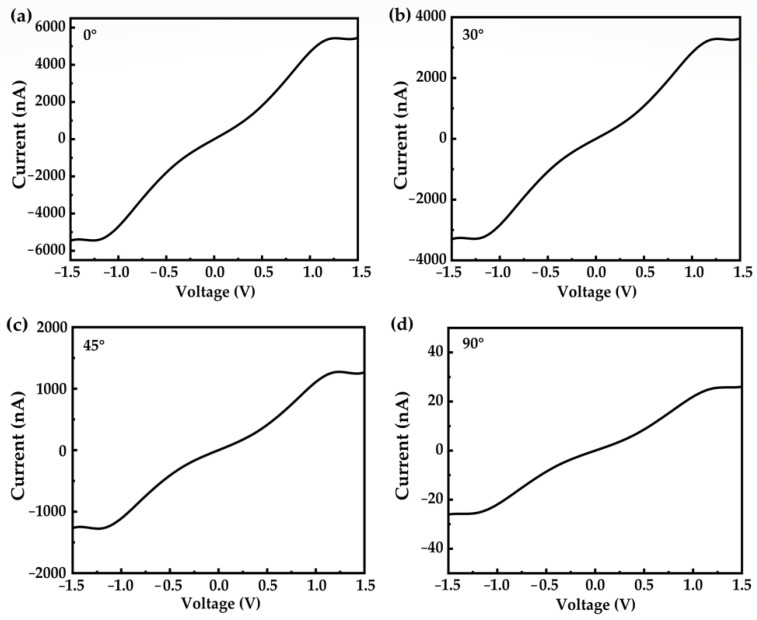
Current–voltage (I–V) characteristic curves of the OPE-3 molecule with different rotation dihedrals of 0° (**a**), 30° (**b**), 45° (**c**), and 90° (**d**).

Considering the fragility of the molecular junction and geometrical variety during the junction formation, we studied the deformation effect on the electron transport [36,37,38]. The current–voltage (I–V) characteristic curves of the molecular junctions with different rotation dihedrals are shown in Figure 4. Compared to the well-conjugated configuration (Figure 4a), rotation reduces the electron transport capacity. To be specific, the conductivity of the OPE-3 molecule with a 0° rotation dihedral is found to be 3650 nA/V. This value is reduced to 2196, 838, and 17 nA/V when the dihedral between the middle and ending benzene is 30, 45, and 90°. It is noted that the dihedral of 90° almost destroys the molecular conjugation, resulting in the device completely losing its transportability (shown in Figure 4d).

**Figure 5:**
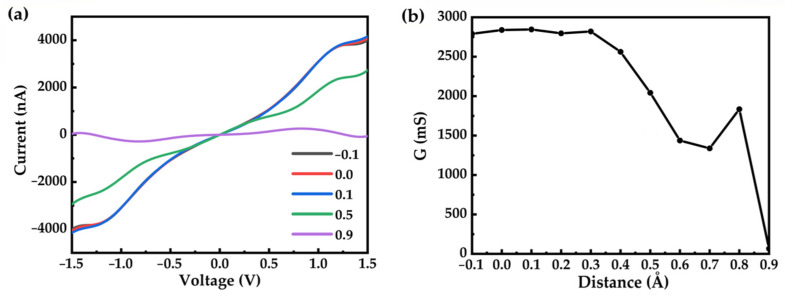
(**a**) I–-V curves of the OPE-3 molecular junction with different electrode distances. (**b**) The relationship between the conductivity curve and the electrode distance.

We further studied the effect of geometrical fluctuation of the electrodes on the electron transport properties. To reproduce the configurational changes of the molecular device caused by the electrodes, we gradually changed the electrode distance from −0.1 to 0.9 Å with an interval of 0.1 Å (minus means stress and plus means tension). We studied the I–V curve, as shown in Figure 5a. We can see that if the electrode distance is depressed or stretched less than 0.4 Å, the conductivity changes very little. However, if the electrode distance is stretched larger than 0.4 Å, the conductivity decreases to a great extent. When the electrode distance is stretched over 1.0 Å, the device completely loses its transport capacity. To systematically study the underlying mechanism of the decrease in conductance when stretching the electrode, we provided detailed information of the molecular junctions (electrode distance, molecular length, and Au–S bond length) in Table S1 in Supplementary Materials. We found that stretching the electrode distance along the Au–S bond results in minimal change in the molecular length (labeled as S–S distance). As a result, the conductance decrease can be attributed to the elongation of the Au–S bond.

## 4. Conclusions

In the present work, we investigate the Raman spectra and charge transport properties of an oligomer phenylene acetylene-3 (OPE-3) molecule trapped in gold junctions based on DFT theoretical calculations. Our investigation shows that injecting charges (−2, −1, 0, +1, +2) into the molecule introduces new Raman peaks located at around 1400 cm^−1^, but only the Raman spectra of the −1 charged molecules agree well with the experimental spectra. With the increase of the external electric field, the new Raman peak of the −1 charged molecule at 1400 cm^−1^ shows gradual blue shifts. We also studied the configurational effect on the Raman spectra and charge transport properties. We found that rotating the benzene groups of the negative OPE-3 molecule by 30, 45, and 90° does not influence the appearance of the new peak at 1400 cm^−1^, while it largely reduces the conductivity. Furthermore, we observed that the conductivity of the molecular device decreases with increasing electrode distance.

## Data Availability

Not applicable.

## Supplementary Materials

The following supporting information can be downloaded at: https://www.mdpi.com/article/10.3390/nano12030571/s1, Figure S1: Comparison of HOMO-LUMO gap for OPE-3 molecule with 0, +1 and −1 charges; Figure S2: Simulated Raman spectra of negatively charged OPE-3 molecules with different directions (X, Y, and Z) and values ((a): 0.1 V/nm; (b): 0.5 V/nm; (c): 1.0 V/nm) of applied electric fields; Figure S3: Comparison of Raman spectra for OPE-3 molecules with different electrode distances ((a): −0.1 Å; (b): 0.9 Å); Figure S4: Energy Levels of neutral (a), positive (b) and negative (c) OPE-3 molecules with rotated dihedral of 30, 45 and 90°. From bottom to top representing HOMO−1, HOMO, LUMO, and LUMO+1; Table S1: Detailed structural information including the molecular junction electrode distance, molecular length (represented by S−S distance) and Au−S bond length during stretching the molecular junction.
