# Supplementary material for: Investigation of SERS and Electron Transport Properties of Oligomer Phenylacetyne-3 Trapped in Gold Junctions
Source: Nanomaterials (Basel). 2022 Feb 7;12(3):571. doi: 10.3390/nano12030571 (PMC8839768; doi:10.3390/nano12030571)
Supplement: Supplementary file 1 [file nanomaterials-12-00571-s001.zip › nanomaterials-1558688-supplementary.pdf]

nanomaterials

Supplementary Materials

Investigation of SERS and Electron Transport Properties of Oligomer Phenylacetyne-3 Trapped in Gold Junctions

Ziyu Liu [1,†], Tingting Hu [1,2,†], Muwafag Osman Adam Balila [1], Jihui Zhang [3], Yujin Zhang [4,*] and Wei Hu [1,*]

[1] School of Chemistry and Chemical Engineering, Qilu University of Technology (Shandong Academy of Sciences), Jinan 250353, China; liuzy0625@163.com (Z.L.); hutingting_1981@163.com (T.H.); moufagbalila533@gmail.com (M.O.A.B.)
[2] Technology College of Chemical Engineering, Qingdao University of Science, Qingdao 266061, China
[3] School of Materials Science and Engineering, Qilu University of Technology (Shandong Academy of Sciences), Jinan 250353, China; beckhamzjh@163.com
[4] School of Electronic and Information Engineering, Qilu University of Technology (Shandong Academy of Sciences), Jinan 250353, China
* Correspondence: zhangyujin@qlu.edu.cn (Y.Z.); weihu@qlu.edu.cn (W.H.)
† These authors contributed equally to this work

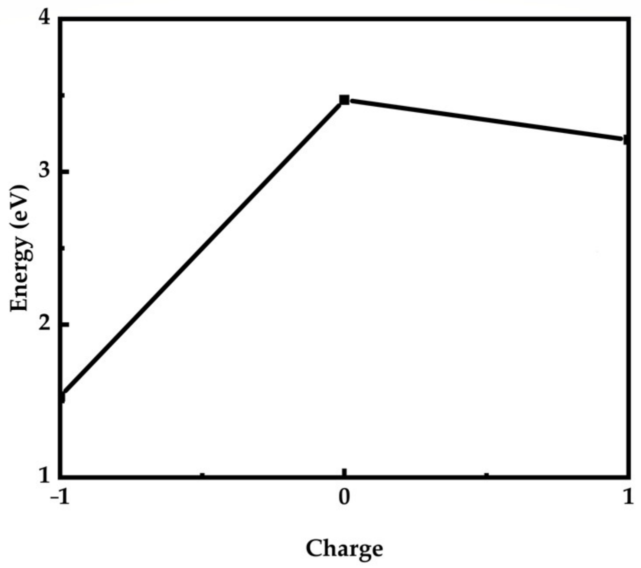

Figure S1. Comparison of HOMO-LUMO gap for OPE-3 molecule with 0, +1 and −1 charges.

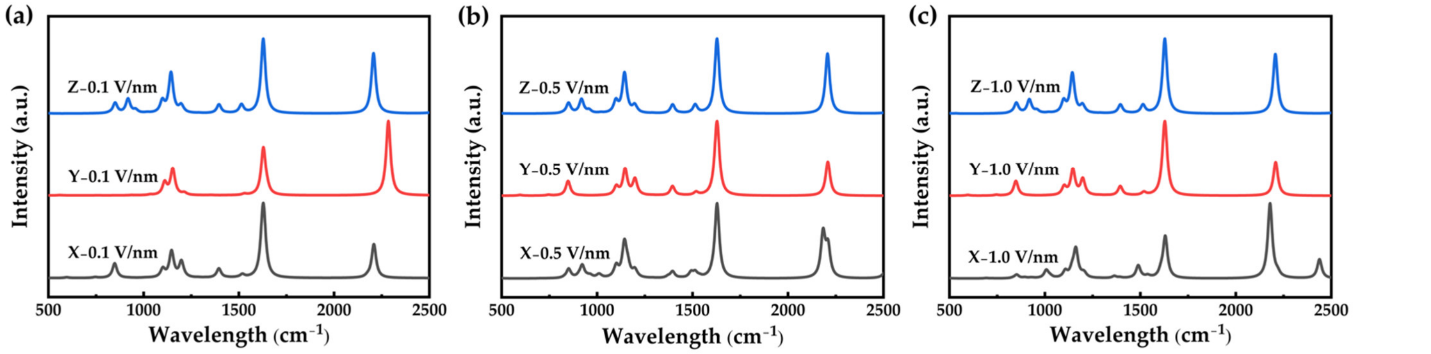

Figure S2. Simulated Raman spectra of negatively charged OPE-3 molecules with different directions (X, Y, and Z) and values ((**a**): 0.1 V/nm; (**b**): 0.5 V/nm; (**c**): 1.0 V/nm) of applied electric fields.

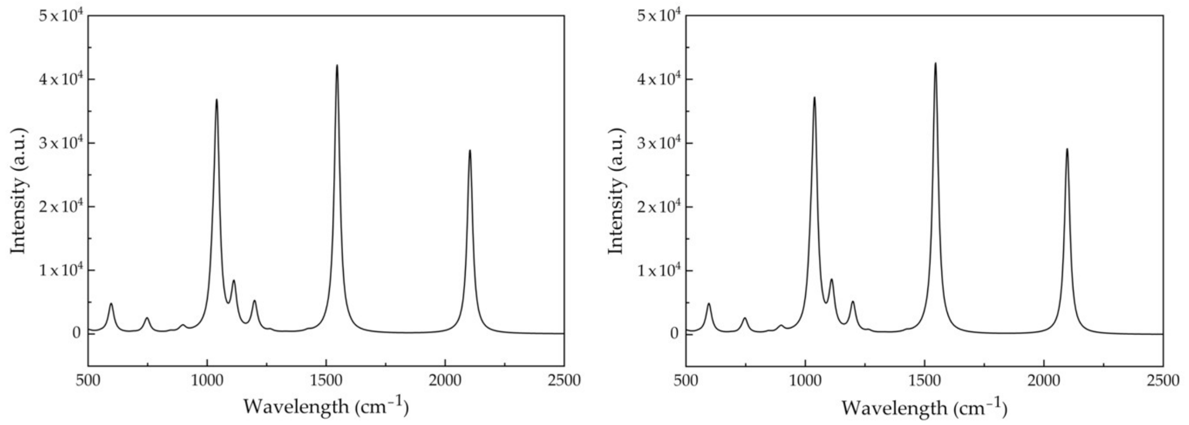

Figure S3. Comparison of Raman spectra for OPE-3 molecules with different electrode distances ((**a**): −0.1 Å; (**b**): 0.9 Å).

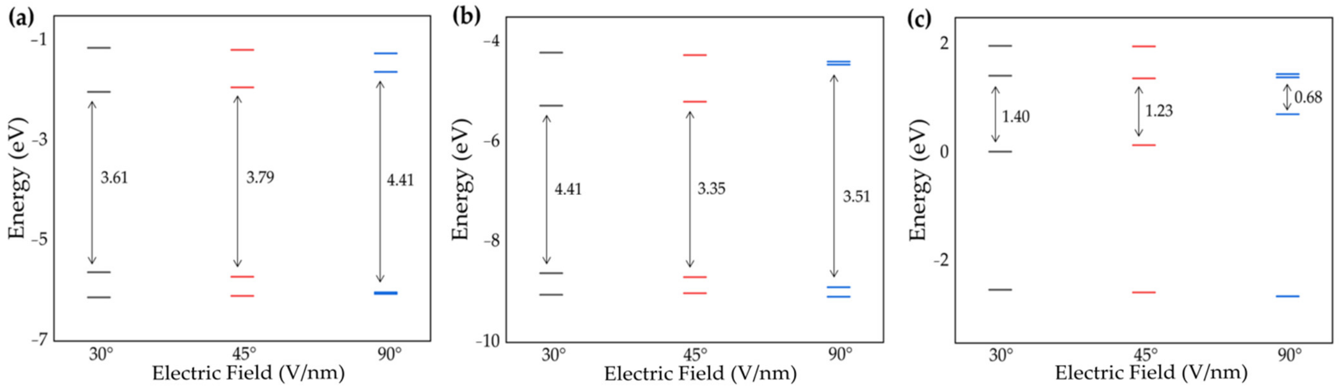

Figure S4. Energy Levels of neutral (**a**), positive (**b**) and negative (**c**) OPE-3 molecules with rotated dihedral of 30, 45 and 90°. From bottom to top representing HOMO−1, HOMO, LUMO, and LUMO+1.

Table S1. Detailed structural information including the molecular junction electrode distance, molecular length (represented by S−S distance) and Au−S bond length during stretching the molecular junction.

Electrode Distance (Å)	Left Au−S Distance (Å)	Right Au−S Distance (Å)	S−S Distance (Å)
−0.1	1.5939	1.587	20.2243
0.0	1.5939	1.587	20.2243
0.1	1.6703	1.6701	20.0997
0.2	1.6703	1.6701	20.0997
0.3	1.6703	1.6701	20.0997
0.4	1.6703	1.6701	20.0997
0.5	1.6656	1.6647	20.1059
0.6	1.6703	1.6701	20.0997
0.7	1.6615	1.6608	20.1111
0.8	1.6592	1.6586	20.1148
0.9	1.6703	1.6701	20.0997
